# Chemotherapy-induced loss of bone and muscle mass in a mouse model of breast cancer bone metastases and cachexia

**Published:** 2019

**Authors:** Brian A. Hain, Haifang Xu, Jenna R. Wilcox, Daniel Mutua, David L. Waning

**Affiliations:** 1 Department of Cellular and Molecular Physiology, Pennsylvania State University College of Medicine, Hershey, PA.; 2 Penn State Cancer Institute, Hershey, PA.

**Keywords:** Cachexia, Chemotherapy, Muscle function, Bone metastases

## Abstract

**Background:**

Chemotherapy used to treat malignancy can lead to loss of skeletal muscle mass and reduced force production, and can reduce bone volume in mice. We have shown that bone-muscle crosstalk is a key nexus in skeletal muscle function and bone homeostasis in osteolytic breast cancer bone metastases. Because chemotherapy has significant negative side effects on bone mass, and because bone loss can drive skeletal muscle weakness, we have examined the effects of chemotherapy on the musculoskeletal system in mice with breast cancer bone metastases.

**Methods and results:**

Six-week-old Female athymic nude mice were inoculated with 10^5^ MDA-MB231 human breast cancer cells into the left ventricle and bone metastases were confirmed by X-ray. Mice were injected with carboplatin at a dose of 60mg/kg once per week starting 4 days after tumor inoculation. Skeletal muscle was collected for biochemical analysis and extensor digitorum longus (EDL) whole muscle contractility was measured. The femur and tibia bone parameters were assessed by microCT and tumor burden in bone was determined by histology. Healthy mice treated with carboplatin lose whole body weight and have reduced individual muscle weights (gastrocnemius, tibialis anterior (TA), and EDL), reduced trabecular bone volume (BV/TV), and reduced EDL function. Mice with MDA-MB-231 bone metastases treated with carboplatin lose body weight, and have reduced EDL function as healthy mice treated with carboplatin. Mice with MDA-MB-231 bone metastases plus carboplatin do have reduced proximal tibia BV/TV compared to carboplatin alone, but carboplatin does reduce tumor burden in bone.

**Conclusions:**

Our data shows that carboplatin treatment, aimed at reducing tumor burden, contributes to cachexia and trabecular bone loss. The muscle atrophy and weakness may occur through bone-muscle crosstalk and would lead to a feed-forward cycle of musculoskeletal degradation. Despite anti-tumor effects of chemotherapy, musculoskeletal impairment is still significant in mice with bone metastases.

## Introduction

Breast cancer is the most common form of cancer in women worldwide and over 250,000 new cases of breast cancer were diagnosed in the United States in 2017 [1,2]. Breast cancer led to an estimated 41,070 total deaths in 2017 [2]. Advanced stage breast cancer becomes invasive when tumor cells metastasize to near and distant tissues throughout the body including lymph nodes, skin, brain, liver, and bones, among others [3]. Breast cancer metastasis to bone leads to increased bone fractures, pain, hypercalcemia, and bone marrow aplasia [4]. Additionally, osteolytic bone metastases cause release of TGFβ stored in the mineralized bone matrix that leads to lower muscle function in mice [5].

Chemotherapy is used to treat cancer and is aimed at reducing and eliminating the cancer cells. Chemotherapy is used in both a neoadjuvant and adjuvant setting [6], and clinically a combination therapy of different chemotherapeutic agents is used in early stage breast cancer to reduce tumor burden while single chemotherapy drugs are commonly used in advanced stages [7]. Carboplatin, a platinum-based chemotherapy drug that interferes with DNA replication and leads to cell death [8], is used as both a neoadjuvant and adjuvant to treat early and late stage breast cancer [9]. However, like many chemotherapy drugs, carboplatin has a range of side-effects including: nephrotoxicity, anemia, nausea, and weakness [10]. Although carboplatin has been approved for use since 1986, there is little literature focusing directly on carboplatin’s effect on bone and muscle mass or muscle function [11]. Clinically patients with bone metastases are typically treated with chemotherapy and an anti-resorptive bone agent to prevent bone loss. While data on carboplatin’s effect on bone is limited, one study indicates that cisplatin, another platinum based therapy, causes decreased trabecular bone volume in the proximal tibia of rats [12]. While chemotherapy treatment is beneficial in reducing tumor burden, it may induce bone turnover and increase circulating cytokines from bone that could cause skeletal muscle dysfunction and actually make bone more susceptible to metastatic lesions.

Chemotherapy has direct effects on host cells and tissues that include loss of lean mass and contractile dysfunction. For instance, cisplatin, adriamycin, and etoposide cause muscle wasting by activing the NF-kB pathway and by inducing high states of oxidative stress [13]. The chemotherapy cocktail Folfiri (5-FU, leucovorin, irinotecan) causes skeletal muscle wasting and muscle dysfunction [14]. Cisplatin has been shown to activate inflammation and ubiquitin-dependent degradation pathways, leading to muscle wasting [15]. Sorafenib, a broad spectrum tyrosine kinase inhibitor, causes muscle wasting via Ca^2+^-dependent and ubiquitin proteolysis [16].

Preserving skeletal muscle mass and bone mass is an important strategy for cancer patients. Cancer patients with loss of lean mass, even in body weight stable individuals, are likely to develop drug-associated toxicity and poor outcomes compared to those that maintain lean mass. In addition, patients with higher amounts of lean mass are more tolerant of higher doses of chemotherapy that ultimately leads to reduced mortality [17, 18]. Experimental strategies to preserve skeletal muscle mass include blocking myostatin signaling, and antagonizing the growth hormone secretagogue receptor (GHSR)-1a, and have been shown to improve survival in mice with cancer cachexia [15, 19, 20]. Preserving bone mass has been achieved using bone anti-resorptive bisphosphonates, such as zoledronic acid, and with denosumab, a receptor activator of nuclear factor-kB (RANK)-ligand (RANKL) inhibitor, both of which block osteoclast-mediated bone resorption [21]. Bisphosphonates and denosumab are both approved for use in patients with cancer metastatic to bone. Maintaining bone health reduces risk of fractures that have direct impacts on survival.

Our data shows that carboplatin causes a decrease in bone and muscle mass, and reduced muscle function in mice. In addition, carboplatin reduced tumor burden in bone of mice with MDA-MB-231 breast cancer bone metastases. While carboplatin has beneficial anti-tumor effects, the negative impact to musculoskeletal health are significant.

## Methods

### Animals

Female athymic nude mice were obtained from Envigo (Madison, WI) at 5 weeks of age. Mice were kept in a quarantine facility and food/water was available ad libitum. All experiments using animals were performed at the Penn State University Milton S. Hershey Medical Center and approved by Penn State University's Institutional Animal Care and Use Committee (IACUC) and have therefore been performed in accordance with the ethical standards laid down in the 1964 Declaration of Helsinki and its later amendments.

### Tumor cell inoculation

Intracardiac inoculation of human MDA-MB-231 breast cancer cells was performed as previously described into six-week old female athymic nude mice [5]. Briefly, MDA-MB-231 were trypsinized, washed, and resuspended in PBS to a final concentration of 10^5^ cells in 100μl. 100,000 cells were inoculated into the left ventricle of each animal by percutaneous injection using a 26-gauge needle. Visualization of bright red blood entering the hub of the needle in a pulsatile fashion indicated a correct position in the left cardiac ventricle. Mice were euthanized at approximately 4-weeks post-inoculation.

### Chemotherapy treatment

Carboplatin (Sigma-C2538) dissolved in PBS or vehicle was injected intraperitoneally (IP) at 60mg/kg once per week. Injections began four days after tumor inoculation or age-matched in the carboplatin treatment only group. All mice were euthanized at the same relative time.

### Radiography

Osteolytic lesions were analyzed by radiography using a Faxitron UltraFocus with Bioptics digital camera (Faxitron Corp., Tucson, AZ) with mice anesthetized under inhaled isoflurane in a prone position. Osteolytic lesion area was quantified using ImageJ v.1.52a software (National Institutes of Health, USA).

### Tissue

Mice were euthanized and the tibialis anterior (TA), soleus, and gastrocnemius were dissected. The extensor digitorum longus (EDL) muscle was dissected for muscle contractility. Muscles were either snap frozen in liquid nitrogen for biochemical analysis or embedded in O.C.T. compound in cryomolds and frozen in liquid nitrogen-cooled 2-methylbutane for histological analysis. The EDL was snap frozen in liquid nitrogen after contractility testing. Muscle tissue was stored at −80°C. Hind limbs were removed with the tibiofemoral joint intact. Excess muscle tissue was carefully removed from the bones, and the distal tibia and proximal femur were cut transversely with a razor in order to expose the medullary cavity. Bones were placed in a perforated cassette and fixed in 10% neutral buffered formalin for 48 hours before being transferred to 70% ethanol and stored until *ex vivo* microCT scan and then decalcified for histology.

### Muscle Function

*Ex vivo* contractility of the EDL muscle was determined as previously described [5]. The EDL was dissected from hind-limbs and stainless steel hooks were tied to the tendons of the muscles using 4-0 silk sutures and the muscles were mounted between a force transducer (Aurora Scientific, Aurora, ON, Canada) and an adjustable hook. The muscles were immersed in a stimulation chamber containing O_2_ (100%) bubbled Tyrode solution (121 mM NaCl, 5.0 mM KCl, 1.8 mM CaCl_2_, 0.5 mM MgCl_2_, 0.4 mM NaH_2_PO_4_, 24 mM NaHCO_3_, 0.1 mM EDTA, 5.5 mM glucose). The muscle was stimulated to contract using a supramaximal stimulus between two platinum electrodes. Data was collected via Dynamic Muscle Control/Data Acquisition (DMC) and Dynamic Muscle Control Data Analysis (DMA) programs (Aurora Scientific). At the start of each experiment the muscle length was adjusted to yield the maximum force. The force–frequency relationships were determined by triggering contraction using incremental stimulation frequencies (0.5 ms pulses at 1–150 Hz for 350 ms at supramaximal voltage). Between stimulations the muscle was allowed to rest for 3 min. At the end of the force measurement, the length (*L_0_*) and weight of the muscle was measured and the muscle was snap frozen in liquid N_2_. To quantify the specific force, the absolute force was normalized to the muscle size, specifically cross-sectional area, calculated as the muscle weight divided by the length using a muscle density constant of 1.056 kg/m^−3^ [22]. The investigators were blinded to treatment of subjects.

### Bone Micro-computed tomography

MicroCT imaging was performed on the distal femur and the proximal tibia using a VIVACT-40 (Scanco Medical AG, Brüttisellen, Switzerland). Scans were acquired using a 17.5 μm^3^ isotropic voxel size, 55 kVp peak X-ray tube potential, 200 ms integration time, and were subjected to Gaussian filtration. Total bone volume and bone volume fraction (BV/TV), trabecular thickness (Tb.Th), trabecular number (Tb.N), trabecular separation (Tb.Sp), connectivity density (Conn.D), cortical bone volume fraction (Ct.BV/TV), and cortical area (Ct.Ar) were evaluated at the distal epiphysis and metaphysis of the femur in a region that spanned 3.5 mm, and at the proximal epiphysis and metaphysis of the tibia in a region that spanned 2.8 mm. A threshold of 160 was used to manually delineate bone from surrounding soft tissue. PuTTy and Xming evaluation software was used for all analyses. The investigators were blinded to treatment of subjects.

### Histology

Transverse muscle sections (10 μm) were sliced with a cryostat microtome (Microm HM 505E; Microm International, Walldorf, Germany) from the proximal belly of the TA and fixed in 4% paraformaldehyde. The muscle sections were incubated with wheat germ agglutinin conjugated to Texas Red-X (ThermoFisher Scientific, USA) diluted in PBS for visualization of muscle fibers under fluorescence microscopy. Images were captured with an AxioCam 503 mono (Zeiss, Germany) using a Photofluor LM75 metal-halide light source (89 North, Williston, VT). The muscle fiber area of ~200 fibers from each muscle was traced and measured using ImageJ v.1.52a software (National Institutes of Health, USA). Bones collected at euthanasia were decalcified by submerging samples in EDTA tetrasodium dissolved in water and glacial acetic acid at room temperature on a stir plate. Bones were left in the solution for seven days and the solution was changed once after 24 hours. After decalcification bones were embedded in paraffin wax for sectioning. Longitudinal, mid-sagittal sections 5.0 μm in thickness from the tibia and femur were cut using a Leica RM2235 manual rotary microtome (Leica). Tissue sections were stained with hematoxylin and eosin (H&E) as follows: Sections were de-waxed with xylenes for three, 3 minute washes. Sections were then rehydrated in descending ethanol (EtOH) solutions (100%, 95%, 70%, 50% EtOH) 3 minutes each followed by Harris hematoxylin (Sigma, HHS16) stain for 1 minute. Sections were gently rinsed in cool, running tap water for 1 minute to de-stain. Sections were then stained with eosin Y solution (Sigma, HT110116) for 2 minutes and immediately dehydrated in ascending EtOH solutions (95% and 100% EtOH) twice for 1 minute. Slides were cleared with three, 1 minute xylenes washes, mounted and cover slipped with Cytoseal XYL (ThermoScientific, 8312-16E). Sections were visualized using an Olympus BX53 microscope and captured at 4x magnification using an Olympus DP73 camera with Olympus cellSens software. Tumor burden was analyzed using ImageJ v.1.52a software (National Institutes of Health, USA).

### Statistical Analysis

Data were analyzed with the use of GraphPad Prism v7.0d software (GraphPad Software, Inc., La Jolla, CA). microCT scans, tissue weight, and muscle cross sectional area analyzed using one-way ANOVA with Tukey’s post-hoc test for multiple comparisons. Differences in osteolytic lesion area and tumor burden between treatment groups were analyzed using Student’s T-test. Differences in body weight, grip strength, and specific force were analyzed using two-way ANOVA. All results were expressed as mean ± SEM, and *p*<0.05 was considered significant.

## Results

*In vivo* treatment with carboplatin causes loss of trabecular bone and lean mass in mice. In order to establish the effect of carboplatin treatment in mice, we treated both healthy mice and mice with bone metastases (human breast cancer MDA-MB-231 cells), with a clinically relevant dose of carboplatin (60mg/kg once per week). Female athymic nude mice were separated into the following groups: 1) non-tumor vehicle control, 2) nontumor treated with carboplatin, 3) MDA-MB-231 bone metastases vehicle treated, and 4) MDA-MB-231 bone metastases treated with carboplatin.

**Figure 1.**
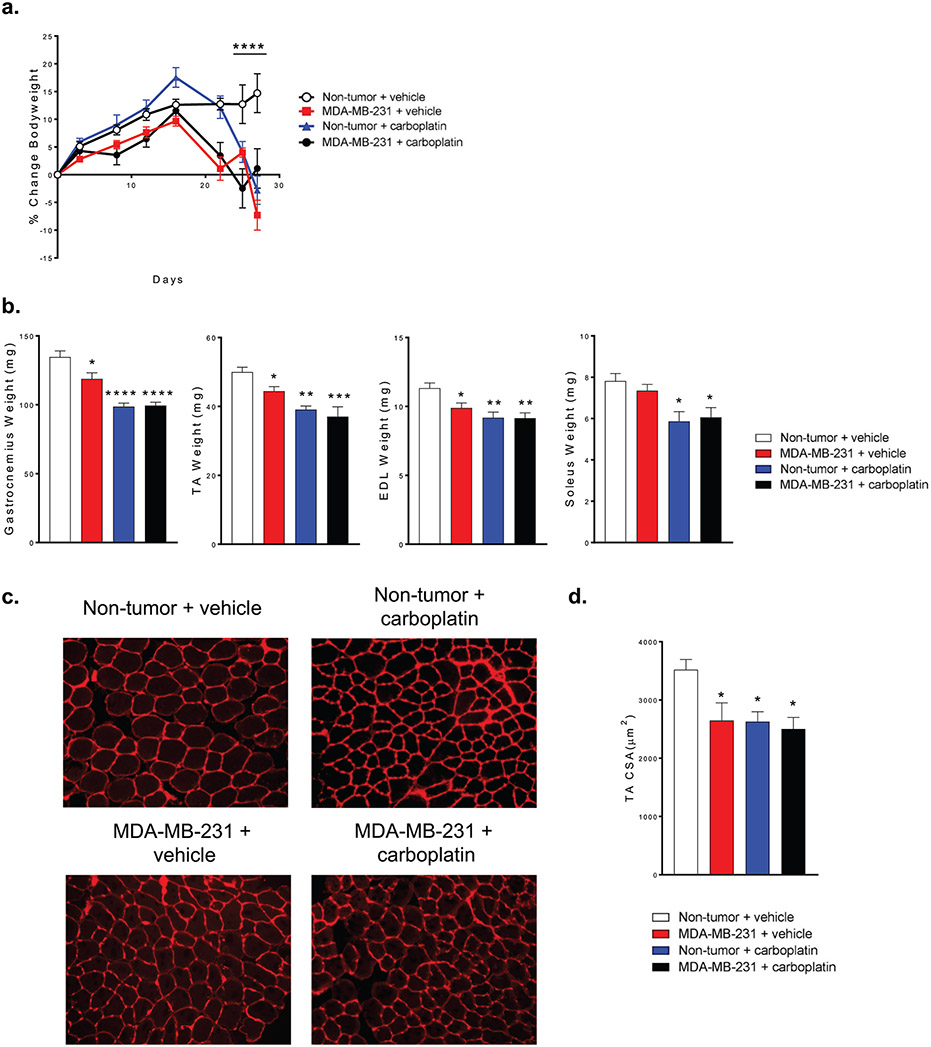
*In vivo* treatment with carboplatin causes loss of lean mass in mice. **(a)** Change in total body weight of mice. **(b)** Change in weight of gastrocnemius, tibialis anterior (TA), extensor digitorum longus (EDL), and soleus muscle. **(c)** Representative cross sections taken from the TA muscle in Non-tumor + vehicle, MDA-MB-231 + vehicle, Non-tumor + carboplatin, and MDA-MB-231 + carboplatin groups. **(d)** Quantitation of cross sectional area (CSA) from TA. (a) Two-way ANOVA (n=7), (b & d) One-way ANOVA with Tukey’s post-hoc test for multiple comparisons (b, n=7 and d, n=5); *p<0.05, **p<0.01, ***p<0.001 compared to control.

Healthy mice treated with carboplatin, mice with MDA-MB-231 bone metastases treated with carboplatin and mice with MDA-MB-231 bone metastases all developed cachexia. The body weight of mice with bone metastases started to decrease at approximately 16-days post-inoculation. Similar kinetics was observed for healthy mice treated with carboplatin. Body weight continued to decrease to a point below their initial body weight (Fig. 1a). The gastrocnemius, tibialis anterior (TA), and extensor digitorum longus (EDL) muscle weights were all significantly lower in all three treatment groups compared to muscles from control animals. The soleus muscle weight was lower in healthy animals treated with carboplatin and in mice with MDA-MB-231 bone metastases treated with carboplatin (Fig. 1b). TA muscles were cryosectioned and treated with wheat germ agglutinin for cross sectional area (CSA) analysis (Fig. 1c). All experimental groups had significantly lower CSA compared to control (Fig. 1d).

**Figure 2.**
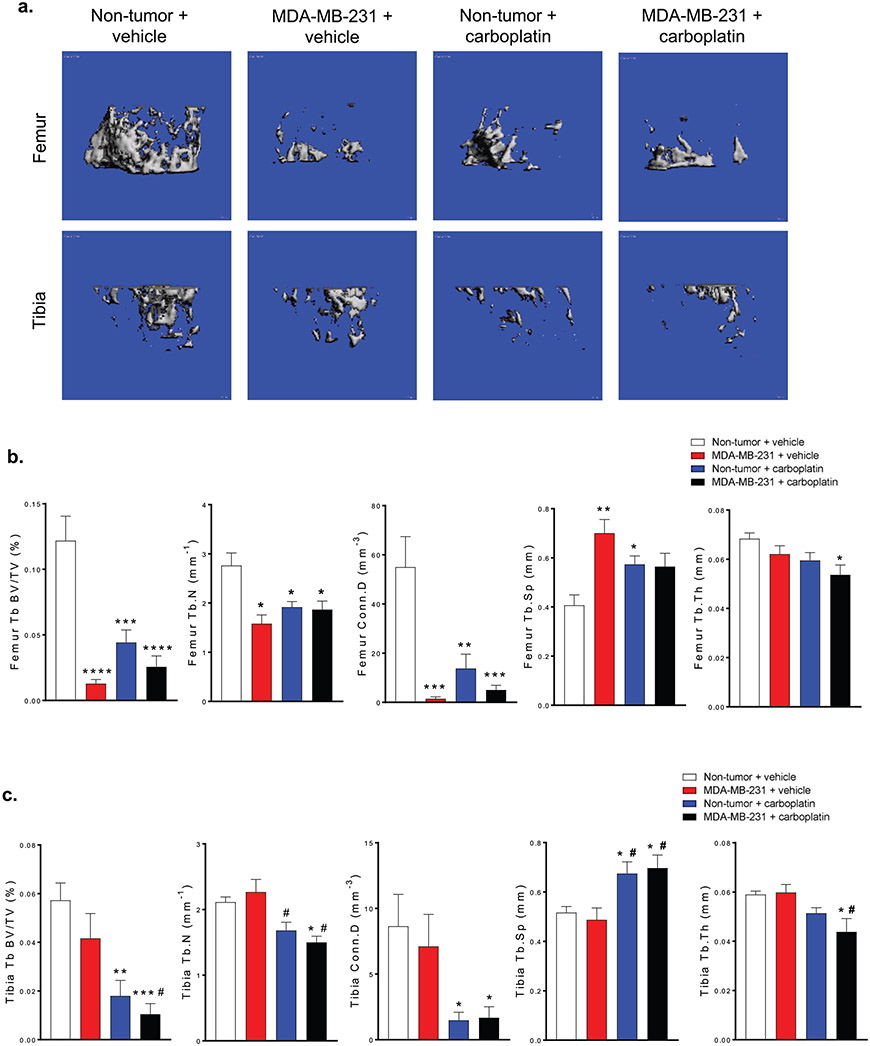
*In vivo* treatment with carboplatin causes loss of trabecular bone in mice. **(a)** Representative microCT scans of the femur and tibia in Non-tumor + vehicle, MDA-MB-321 + vehicle, Non-tumor + carboplatin, and MDA-MB-231 + carboplatin groups. Quantitation of Tb. BV/TV, Tb.N, Conn.D, Tb.Sp, and Tb.N of the **(b)** femur and **(c)** tibia for Non-tumor + vehicle, MDA-MB-321 + vehicle, Non-tumor + carboplatin, and MDA-MB-231 + carboplatin groups, (b & c) One-way ANOVA with Tukey’s post-hoc test for multiple comparisons (n=7), *p<0.05, **p<0.01, ***p<0.001, ****p<0.0001 compared to control group. #p<0.05 compared to MDA-MB-231 + vehicle.

The distal femur and proximal tibia of the hind limbs from all four groups were scanned using microCT and analyzed with PuTTy and Xming evaluation software (Fig. 2a). In the femur, trabecular bone volume (Tb.BV/TV), trabecular number (Tb.N), and connectivity density (Conn.D) were reduced significantly in all groups compared to control while in the tibia only the healthy mice treated with carboplatin and mice with MDA-MB-231 bone metastases and carboplatin were significantly affected compared to control (Fig 2b). Femoral trabecular separation (Tb.Sp) increased in the mice with MDA-MB-231 bone metastases and healthy mice treated with carboplatin, but were not significant in the mice with MDA-MB-231 bone metastases and treated with carboplatin. Tibia Tb.Sp increased in both the healthy mice treated with carboplatin and the mice with MDA-MB-231 bone metastases compared to control and MDA-MB-231 bone metastases groups. Finally, in both the femur and tibia, trabecular thickness (Tb.Th) was lower in the mice with MDA-MB-231 bone metastases and treated with carboplatin (Fig. 2c).

These data show that carboplatin alone causes loss of lean skeletal muscle and bone mass. In addition, the loss of lean mass and bone mass in mice with MDA-MB-231 bone metastases is not prevented by the anti-tumor effects of carboplatin.

**Figure 3.**
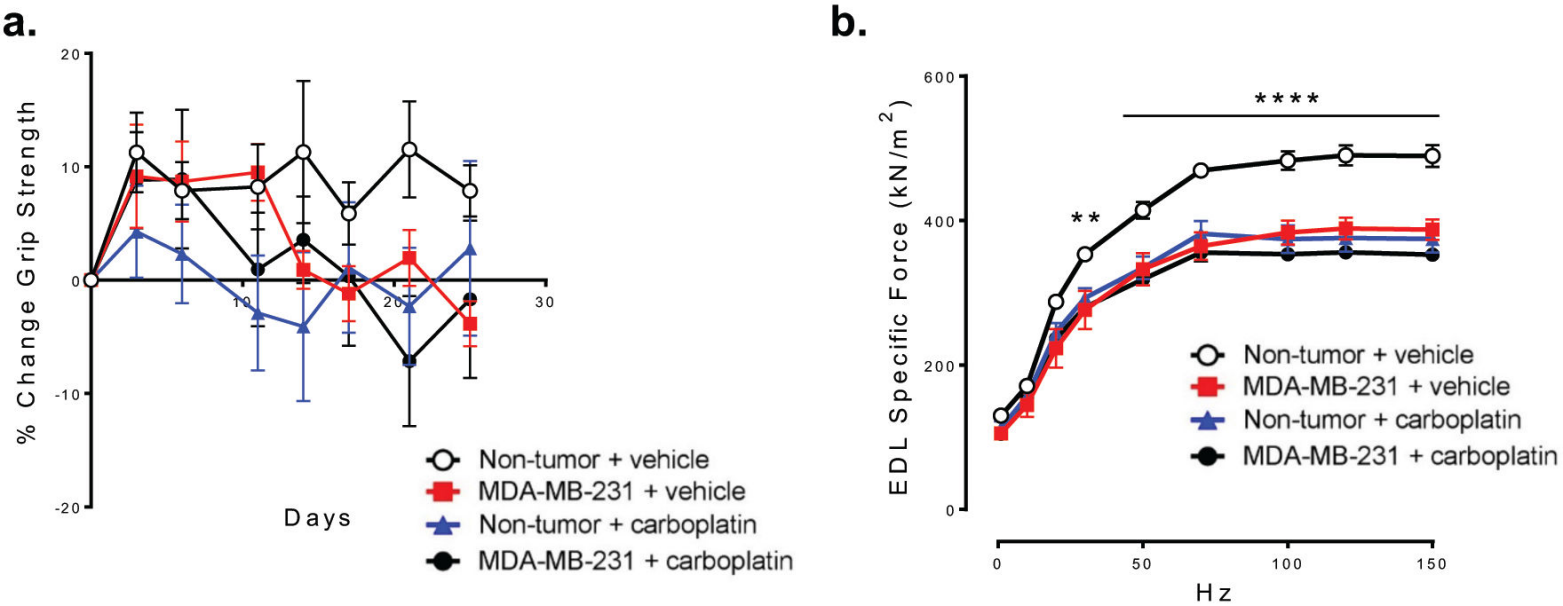
*In vivo* treatment with carboplatin causes skeletal muscle dysfunction. **(a)** Forelimb grip strength in Non-tumor + vehicle, MDA-MB-321 + vehicle, Non-tumor + carboplatin, and MDA-MB-231 + carboplatin groups, **(b)** Whole muscle contractility of the EDL muscle (specific force) from Non-tumor + vehicle, MDA-MB-321 + vehicle, Non-tumor + carboplatin, and MDA-MB-231 + carboplatin groups, (a & b) Two-way ANOVA with Tukey’s post-hoc test for multiple comparisons (n=10 per group), **p<0.01, ****p<0.0001 compared to Non-tumor + vehicle.

*In vivo* treatment with carboplatin causes skeletal muscle dysfunction. We next wanted to test skeletal muscle function. We measured forelimb grip strength longitudinally. We found no significant difference in grip strength between any of the groups (Fig. 3a). Because of the high variability in grip strength testing and the potential for habituation, we examined muscle contractility directly by whole muscle isometric force measurements of excised EDL muscle. The specific force (raw force corrected for size and weight differences) was significantly lower in mice with MDA-MB-231 bone metastases and healthy mice treated with carboplatin compared to control mice. Mice with MDA-MB-231 bone metastases and treated with carboplatin also had reduced specific force that was no worse than either experimental single effect group (Fig. 3b). These data show that carboplatin alone causes reduced muscle function in healthy mice and does not reverse muscle dysfunction in mice with MDA-MB-231 bone metastases.

**Figure 4.**
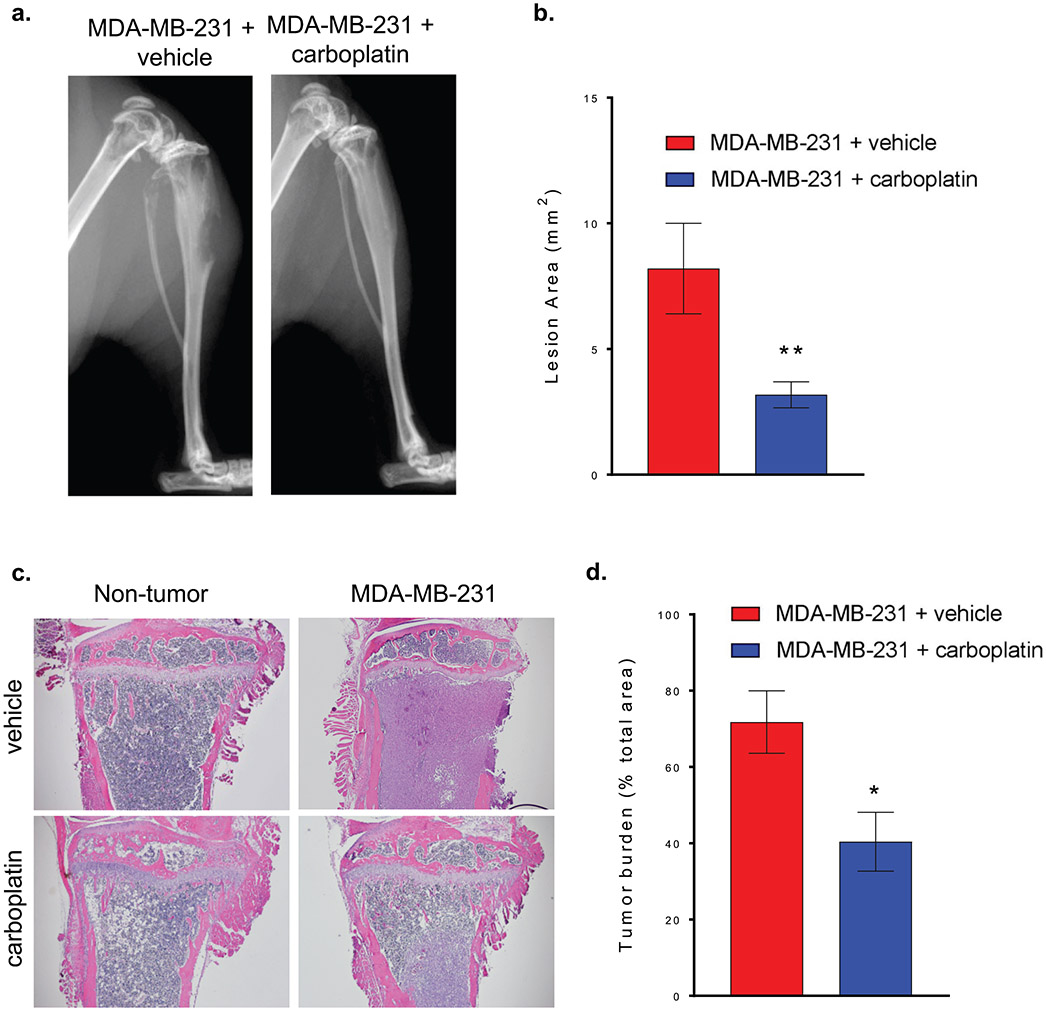
Carboplatin reduced osteolytic lesion area and tumor burden. **(a)** Representative X-ray images of hind limbs of mice 4 weeks after tumor inoculation and tumor inoculation with carboplatin treatment. **(b)** Quantitation of whole body osteolytic lesion area in MDA-MB-231 + vehicle and MDA-MB-231 + carboplatin groups. **(c)** Representative images of bone histology sections stained with H&E from Non-tumor + vehicle, MDA-MB-321 + vehicle, Non-tumor + carboplatin, and MDA-MB-231 + carboplatin groups. **(d)** Quantitation of tumor burden area in MDA-MB-231 + vehicle and MDA-MB-231 + carboplatin groups, (b & d) Student’s T-test (b, n=10 and d, n=5), #p<0.05, ##p<0.01 compared to MDA-MB-231 + vehicle.

Carboplatin reduced osteolytic lesion area and tumor burden. To determine the impact of carboplatin on tumor cells in mice with MDA-MB-231 bone metastases, we measured osteolytic lesion area and tumor burden. Mice with MDA-MB-231 bone metastases develop osteolytic lesions. Osteolytic lesion area was quantified by X-ray at euthanasia (Fig 4a). Mice with MDA-MB-231 bone metastases treated with carboplatin had lower osteolytic lesion area by approximately 58% (Fig 4b). Hind limbs from all groups were decalcified, embedded in paraffin, sectioned longitudinally, and stained with H&E (Fig 4c). Tumor burden in bone was lower in mice treated with carboplatin (Fig 4d).

**Figure 5.**
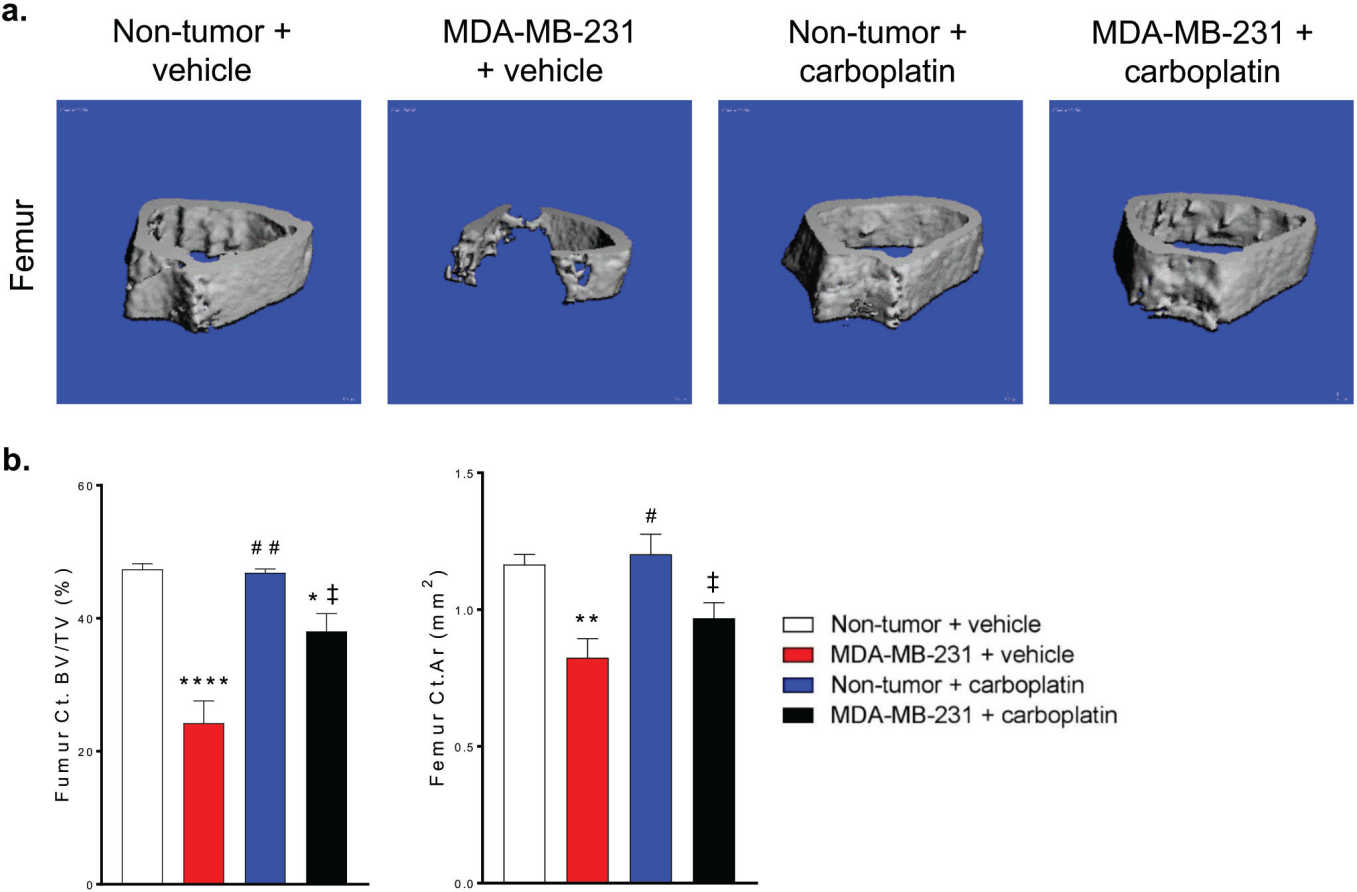
Carboplatin reduced tumor cell-induced loss of cortical bone. **(a)** Representative microCT scans of the femur from Non-tumor + vehicle, MDA-MB-321 + vehicle, Non-tumor + carboplatin, and MDA-MB-231 + carboplatin groups. **(b)** Quantitation of Ct. BV/TV and Ct.Ar. (b) One-way ANOVA with Tukey’s post-hoc test for multiple comparisons, (n=7), *p<0.05, **p<0.01, ****p<0.0001 compared to Non-tumor + vehicle group. #p<0.05, ##p<0.01 compared to MDA-MB-231 + vehicle group. ‡p<.05 compared to Non-tumor + carboplatin group.

Carboplatin reduced tumor cell-induced loss of cortical bone. Mice with MDA-MB-231 bone metastases develop osteolytic lesions which often appear as complete loss of trabecular bone and loss of cortical bone near the metaphysis. We examined the femur cortical bone in this region by microCT. Mice with MDA-MB-231 bone metastases and treated with carboplatin had more cortical bone than vehicle treated mice. Healthy mice treated with carboplatin did not have a change in cortical bone parameters at this site compared to control mice (Fig. 5a-b). These data show that carboplatin does reduce the impact of MDA-MB-231 tumor cells on the skeleton.

## Discussion

Chemotherapy treatment is a necessary neoadjuvant and adjuvant therapy in patients with cancer to reduce or eliminate tumor cells. However, chemotherapy side-effects can be severe and negatively affect quality of life during treatment including a prevalence of physical weakness [23]. Carboplatin is often used to treat triple negative breast cancer in both early and late stages. Advanced breast cancer metastasis to bone is common, however there is little information on the effect of carboplatin on muscle or bone [7] and no studies have looked at overall impact of carboplatin and bone metastases on the musculoskeletal system. In this study, a mouse model of bone metastasis coupled with carboplatin treatment was used to examine the combined effects on muscle and bone.

Carboplatin induces trabecular bone loss but does not exacerbate bone metastases-induced trabecular bone loss. It is well established that patients with bone metastases from a variety of primary cancers including lung [24], breast [25], prostate [26], and others [27] develop cachexia. Our data shows that a clinically relevant dose of carboplatin in healthy mice induces trabecular bone loss (decrease in Tb.BV/TV, Tb.N, and Conn.D and an increase in Tb.Sp in the distal femur and proximal tibia of mice treated with carboplatin). These findings are supported by studies that reported combination chemotherapy treatment of 5-fluorouracil, cyclophosphamide, and epirubicin caused trabecular bone loss in rats [28] and that Folfiri (5-fluorouracil, leucovorin and irinotecan) caused loss of muscle and bone mass [14]. Additionally, data suggests that cisplatin, another platinum-based chemotherapy drug, induces a decrease in Tb.BV/TV in the proximal tibia of rats [12]. In mice with bone metastases, trabecular bone in the femur was also reduced, likely due to an increase in osteoclast activity mediated by tumor cells [29]. Mice with bone metastases treated with carboplatin did not exhibit greater bone loss than healthy mice treated with carboplatin.

Carboplatin causes muscle atrophy but does not exacerbate cancer cachexia. Our data show that carboplatin treatment alone is sufficient to induce a significant decrease in body mass including reduction in gastrocnemius, TA, soleus, and EDL muscle weight, as well as a reduction in TA CSA. Additionally, carboplatin reduced the specific force in the EDL. There is no data in the literature that has established the effect of carboplatin treatment alone on the regulation of muscle mass or function. There is, however, data that has shown cisplatin treatment causes loss of body weight and muscle mass, in mice and rats [30, 31]. Cisplatin increases mRNA expression of the ubiquitin E3 ligases muscle RING finger-1 (MuRF1), muscle atrophy F-box (MAFbx), the transcription factor forkhead box O3 (FOXO3), and nuclear factor of κB (NF-κB) activity, all of which are instrumental in muscle protein degradation signaling [30, 31]. Phosphorylation of Akt was significantly decreased in muscles of mice treated with cisplatin which is a marker of decreased mammalian target of rapamycin (mTOR) dependent protein synthesis [30]. While increases in rates of protein degradation and decreases in rates of protein synthesis in chemotherapy treated muscles explain the overall loss of muscle mass, the upstream activation of these pathways may be multifaceted. One study using cisplatin treated C2C12 myotubes, showed that mitochondrial dysfunction occurs and contributes to muscle atrophy [32]. Since mitochondrial dysfunction causes oxidative stress and increases protein degradation pathways in skeletal muscle [33], this may be one of the contributing factors to the loss of muscle mass and force production in mice treated with carboplatin. However, carboplatin induces trabecular bone loss and as previously described, bone loss leads to increased circulating TGF-β that leads to muscle weakness [5]. Because carboplatin treatment induces trabecular bone loss, upstream muscle atrophy signaling cascades such as NF-κB and FOXO may also be activated, in part, by cytokines originating from bone. Interestingly, the muscle mass and specific force of mice with bone metastases treated with carboplatin did not show an increased degree of muscle atrophy or dysfunction compared to mice with bone metastases treated with vehicle. This may be due to overlapping downstream transcriptional pathways initiated by cytokine circulation from bone resorption in both carboplatin treatment and bone metastases.

Bone metastases cause osteolytic lesions by disrupting the balance between bone resorption and bone formation [34, 35]. Breast cancer bone metastases increase osteoclast activity which leads to greater bone resorption [36]. Eventually osteoclast activity is so great that lesions form in the bone which are visible by X-ray. While carboplatin treatment alone caused trabecular bone loss alone, there was no loss of cortical bone. In mice with MDA-MB-231 bone metastases, osteolytic lesions cause loss of both trabecular and cortical bone. Carboplatin reduced tumor burden in these mice and prevented loss of cortical bone.

In conclusion, our findings describe the effect of carboplatin treatment on bone and muscle in mice with and without breast cancer bone metastases. Our data shows that carboplatin causes a loss of muscle and bone, but does not exacerbate cachexia in mice with bone metastases. Carboplatin treatment is effective at reducing tumor burden in mice with bone metastases, but does have significant negative side-effects on the musculoskeletal system.
